# Supplementary material for: Study on Doxorubicin Loading on Differently Functionalized Iron Oxide Nanoparticles: Implications for Controlled Drug-Delivery Application
Source: Int J Mol Sci. 2023 Feb 24;24(5):4480. doi: 10.3390/ijms24054480 (PMC10002596; doi:10.3390/ijms24054480)
Supplement: Supplementary file 1 [file ijms-24-04480-s001.zip › ijms-2206754-supplementary.pdf]

International Journal of *Molecular Sciences*

MDPI

Supplementary Materials

Study on doxorubicin loading on differently functionalized iron oxide nanoparticles: implications for controlled drug-delivery application

Vladislav R. Khabibullin [1,2], Margarita R. Chetyrkina [3], Sergei I. Obydennyy [4,5], Sergey V. Maksimov [1], Gennady V. Stepanov [2] and Sergei N. Shtykov [6,*]

[1] Chemistry Department, M.V. Lomonosov Moscow State University, d. 1, str. 3, Lenin Hills, Moscow, GSP-1 V-234, 119991, Moscow, Russia
[2] State Scientific Center of the Russian Federation, Joint Stock Company "State Order of the Red Banner of Labor Research Institute of Chemistry and Technology of Organoelement Compounds", 105118 Moscow
[3] Skolkovo Institute of Science and Technology, Nobel Str. 3, 143026, Moscow, Russia
[4] Dmitry Rogachev National Medical Research Center Of Pediatric Hematology, Oncology and Immunology, Samory Mashela, 1, 117198, Moscow, Russia
[5] Center for Theoretical Problems of Physicochemical Pharmacology, Leninsky Prospekt, 38, 119334, Moscow, Russia
[6] Department of Analytical Chemistry and Chemical Ecology, Institute of Chemistry, Saratov State University, 410012, Saratov, Russia
* Correspondence: shtykovsn@mail.ru (S.N.S); vladhab1995@gmail.com (V.R.K.); Tel.: +7-8452-51-64–11 (S.N.S)

Figures:

Figure S1. X-ray diffraction pattern of magnetite nanoparticles

Figure S2. SEM-image (a) and TEM-images (b and c) of pure magnetite nanoparticles (Sample Fe_3O_4)

Figure S3. TEM images of IONs, where (a) and (b) are unmodified magnetite Fe_3O_4, (c) and (d) are PSS-modified magnetite Fe_3O_4@PSS, and (e) and (f) refer to PEI-modified magnetite Fe_3O_4@PEI.

Figure S4. Recorded magnetization curves: 1 – Fe_3O_4@C; 2 – Fe_3O_4@PEI; 3 – Fe_3O_4

Figure S5. Scheme for assessing the colloidal stability of dispersions

Figure S6. Hydrodynamic size distribution of IONs, where the red line is pH 5, the blue line is pH 7.4.

Figure S7. Payload release from drug-loaded nanosorbents in buffer solution with (a) pH 7.4 and (b) pH 5.0 as a function of time for the first 60 minutes (n = 3, P = 0.95).

Tables:

Table S1. Selection of polymeric coatings for the functionalization of magnetite for the purpose of doxorubicin delivery.

Table S2. Comparison of the sorption of doxorubicin by different types of sorbents

Procedures

Evaluation of the colloidal stability of dispersions

Figures

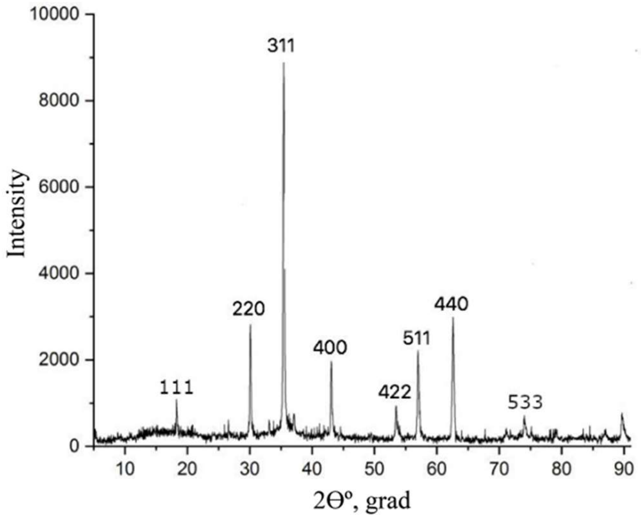

Figure S1. X-ray diffraction pattern of magnetite nanoparticles (Sample Fe_3O_4)

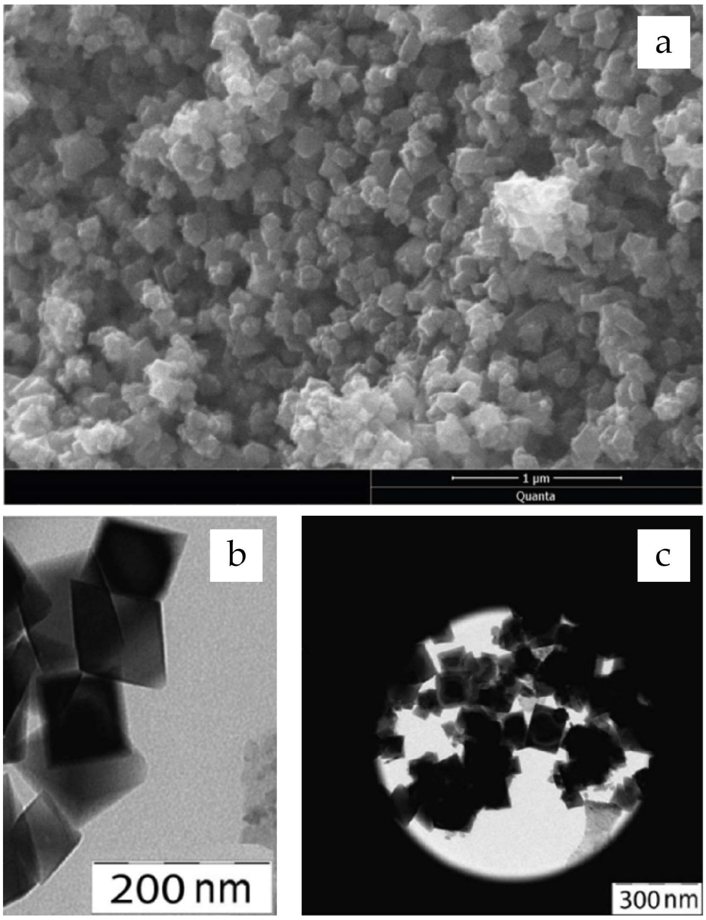

Figure S2. SEM-image (a) and TEM-images (b and c) of pure magnetite nanoparticles (Sample Fe_3O_4)

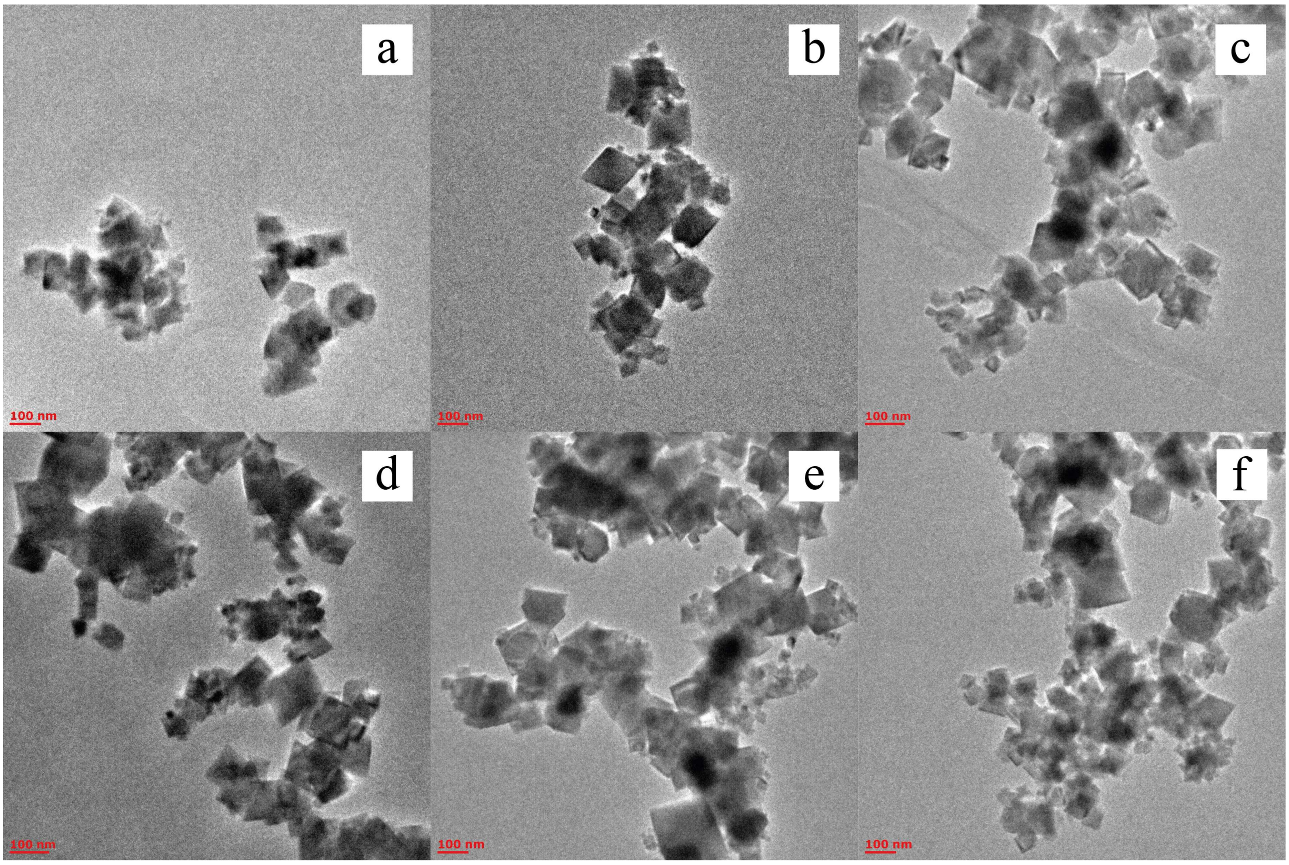

Figure S3. TEM images of IONs, where (a) and (b) are unmodified magnetite Fe_3O_4, (c) and (d) are PSS-modified magnetite Fe_3O_4@PSS, and (e) and (f) refer to PEI-modified magnetite Fe_3O_4@PEI.

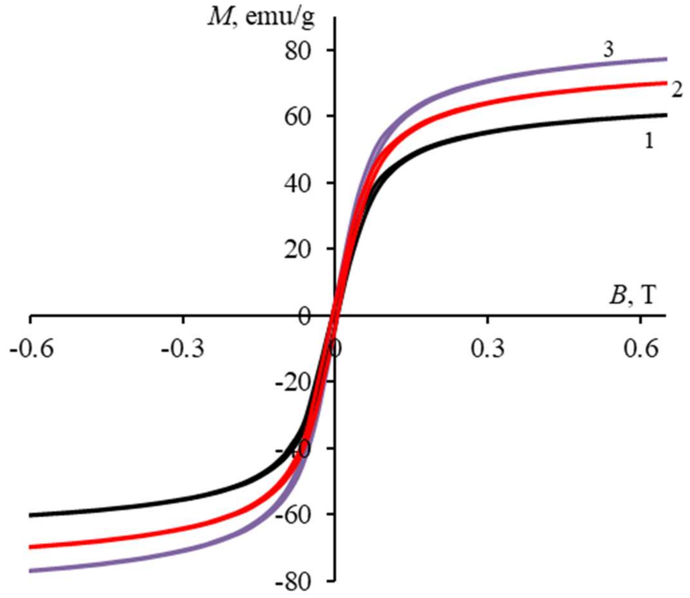

Figure S4. Recorded magnetization curves: 1 – Fe_3O_4@Carb; 2 – Fe_3O_4@PEI; 3 – Fe_3O_4

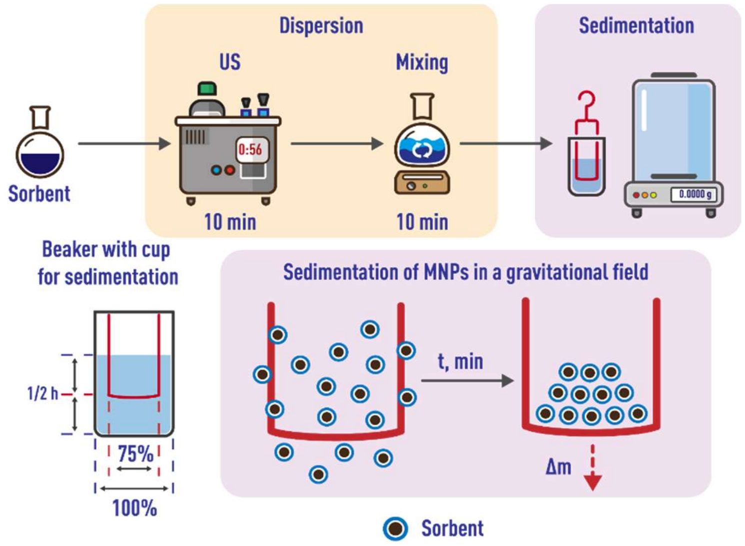

Figure S5. Scheme for assessing the colloidal stability of dispersions

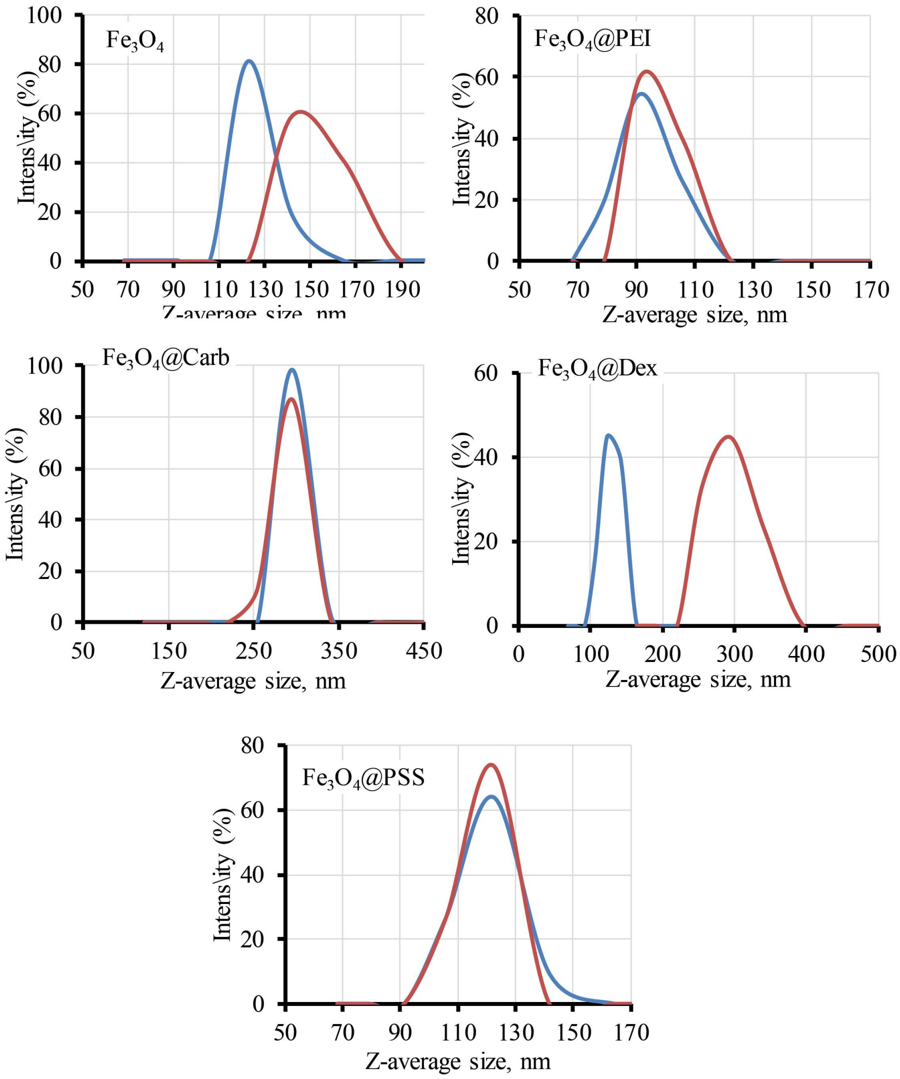

Figure S6. Hydrodynamic size distribution of IONs by DLS, where the red line is pH 5, the blue line is pH 7.4.

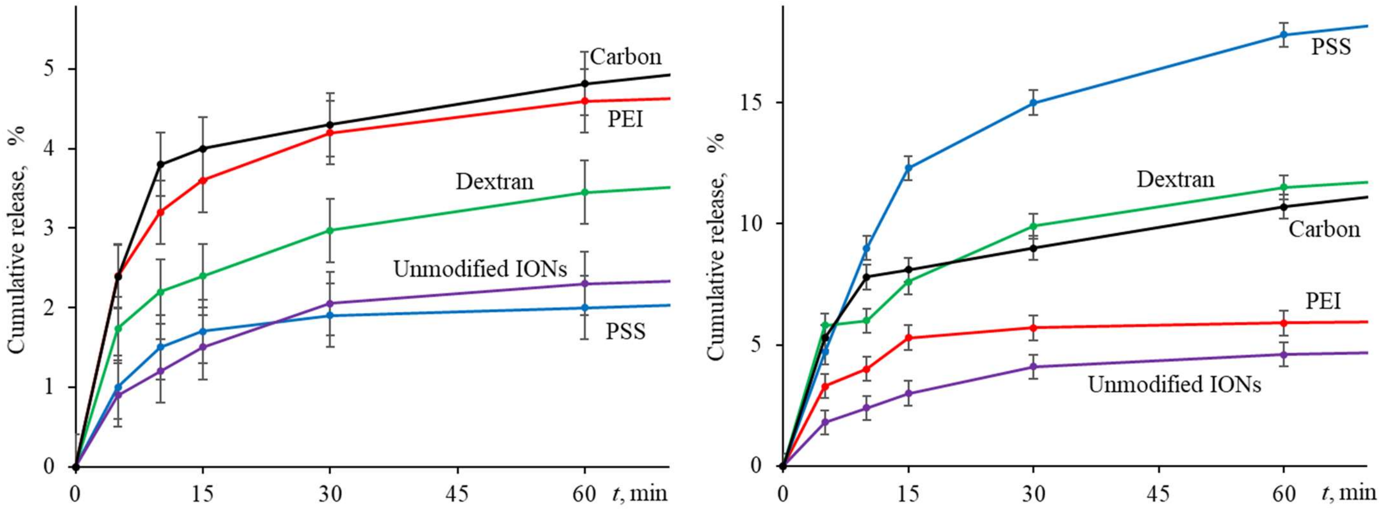

(**a**) (**b**)

Figure S7. Payload release from drug-loaded nanosorbents in buffer solution with (a) pH 7.4 and (b) pH 5.0 as a function of time for the first 60 minutes (n = 3, P = 0.95).

Tables

Table S1. Selection of polymeric coatings for the functionalization of magnetite for the purpose of doxorubicin delivery.

Modifier	Size of IONs, nm	Active surface group	Ref.
PEG – APTES	10, 17	OH; NH_2; Si-O-C	[1]
PEG – phospholipids	22.9	OH; C=O; POOH; P-O-C; C-O-C	[2]
Carboxylated PEG	10	COOH; OH	[3]
PEG – PAMAM	10 – 15	OH; C=O; NH; NH_3^+	[4]
PNIPAAm – MAA	20–75	COOH; C=O; NH	[5]
P(NIPA-PEGMEA)-*b*-PAA	20–25	COOH; C=O; C-O-C; NH; C=S	[6]
PAA	6	COOH	[7]
Alginate	18; 91	COOH; OH; C-O-C	[8]
Heparin	125 ± 10;<br>10, 42;	SO_3H ; OH; COOH; C-O-C; NH	[9,10]
APTS–PAMAM	45 ± 10	OH; C=O; NH; NH_2; NH_3^+	[11]
APTS–arginine	9.2; 9.8	COOH; NH_2 NH; =NH_2	[12]
Chitosan	10; 69	OH; NH_2	[13]

Table S2. Comparison of the sorption of doxorubicin by different types of sorbents

Sorbent		Sorption				Ref.
**Type**	**The average size d_{av} by TEM, nm**	**Sorption capacity q, $mg_{DOX}\cdot g^{-1}_{sorb}$**	**Time, h**	**pH**	**Medium**	
Activated carbon	65	90.87	1	5	-	[14]
Zr-MOF	70	630.48	1,66	6	-	[15]
Se/CTAB- Protein NP	33.3	61%	24	7.4	-	[16]
Au-polysaccharide NP	7.47	$1.66\cdot10^3$ (99.27 %)	1	5.0	-	[17]
Mesoporous Si microspheres	420	20	24	-	water	[18]
Mesoporous carbon	-	374.3	12	-	water	[19]
Fe_3O_4/SiO_2	200	$28\cdot10^3$	24	-	water	[20]
Hydroxyapatite	$(1\text{-}1.5)\cdot10^3$	15.5	24	-	water	[21]
GS-Au-Fe_3O_4	120	-	48	-	water	[22]
Liposome-Fe_3O_4	70-100	-	4	6.2	citrate	[23]
Fe_3O_4@ OA, Tw, PEG	10-200	590-868	-	8.5	SBB	[24]
Fe_3O_4@Au/PPy NP	150-200	-	~12	-	PBS	[25]
Fe3O4@Si-Carrageenan	10	123	24	6	PBS	[26]
Carbon nanotubes	200-800*	$1.03\cdot10^3$	2	7.4	PBS	[27]
Nanotubes grafted with β-cyclodextrin	100-200*, 50-70	48.12	24	7.4	PBS	[28]
Ag-PEG NP	51	882.7	24	7.4	PBS	[29]
Fe_3O_4/SiO_2 NP	~18.1	69	24	7.4	PBS	[30]
Graphene oxide	>µm	-	24	7.4	PBS	[31]
Fe_3O_4@C/ZnO-FA	300-400	64.35	24	7.4	PBS	[32]
Fe_3O_4 -PVCL-co-PAA	70x100	9.6-91.1	72	7.4	PBS	[33]
Fe_3O_4@SiO_2 –Glu NP	30-50	36.8	>80	7.4	PBS	[34]
Nanofiber	330	40.70	2	8	PBS	[35]
Fe_3O_4 NP	150	45	0.75	7.4	PBS	Our study
Fe_3O_4/DEC NP	130	63				
Fe_3O_4/C NP	290	151				
Fe_3O_4/PSS	118	325				
Fe_3O_4/PEI	90	691				

*nanotube length, SBB - sodium borate buffer, PBS -phosphate-buffered saline

Procedures

Evaluation of the colloidal stability of dispersions

An important characteristic of NP dispersions is its stability over time from aggregation and sedimentation. Here, to assess the sedimentation stability, the method of sedimentation in a gravitational field was used [36]. Briefly, 30 ml of an aqueous solution of magnetite at a concentration of 50 $mg\cdot mL^{-1}$ was kept for 10 minutes first in an ultrasonic bath and then stirred in a vertical rotator. The solution was quantitatively transferred into a glass beaker with a capacity of 50 ml, into which a metal cup suspended from an analytical balance was immersed (Figure S5). Further, with the help of a PC connected to an analytical balance, the mass of the cup was recorded in time and built by nodding in differential form ($\Delta m/\Delta t$ from t). The experiment continued until the mass of the cup became constant for 3 minutes.

The dependence of the cup mass on time in the differential form $\Delta m/\Delta t$ on t was constructed using the following equation:

$$\frac{\Delta m}{\Delta t} = \frac{m(t_{i+1})-m(t_i)}{t_{i+1}-t_i}, \quad (1)$$

where $m(t_i)$ is the mass of the cup at time t_i (ms), $m(t_{i+1})$ is the mass of the cup at time t_{i+1} (ms). Thus, the peak of the differential curve corresponds to 0.5 of the total mass of the sorbent settled during the experiment, and the time in this case is the average time of sedimentation.

References

1. Javid, A.; Ahmadian, S.; Saboury, A.A.; Kalantar, S.M.; Rezaei-Zarchi, S.; Shahzad, S. Biocompatible APTES–PEG Modified Magnetite Nanoparticles: Effective Carriers of Antineoplastic Agents to Ovarian Cancer. *Applied Biochemistry and Biotechnology* **2014**, *173*, 36-54, doi:10.1007/s12010-014-0740-6.
2. Shen, C.; Wang, X.; Zheng, Z.; Gao, C.; Chen, X.; Zhao, S.; Dai, Z. Doxorubicin and indocyanine green loaded superparamagnetic iron oxide nanoparticles with PEGylated phospholipid coating for magnetic resonance with fluorescence imaging and chemotherapy of glioma. *Int J Nanomedicine* **2019**, *14*, 101-117, doi:10.2147/IJN.S173954.
3. Liang, P.C.; Chen, Y.C.; Chiang, C.F.; Mo, L.R.; Wei, S.Y.; Hsieh, W.Y.; Lin, W.L. Doxorubicin-modified magnetic nanoparticles as a drug delivery system for magnetic resonance imaging-monitoring magnet-enhancing tumor chemotherapy. *Int J Nanomedicine* **2016**, *11*, 2021-2037, doi:10.2147/ijn.S94139.
4. Nigam, S.; Chandra, S.; Newgreen, D.F.; Bahadur, D.; Chen, Q. Poly(ethylene glycol)-Modified PAMAM-Fe3O4-Doxorubicin Triads with the Potential for Improved Therapeutic Efficacy: Generation-Dependent Increased Drug Loading and Retention at Neutral pH and Increased Release at Acidic pH. *Langmuir* **2014**, *30*, 1004-1011, doi:10.1021/la404246h.
5. Akbarzadeh, A.; Samiei, M.; Joo, S.W.; Anzaby, M.; Hanifehpour, Y.; Nasrabadi, H.T.; Davaran, S. RETRACTED ARTICLE: Synthesis, characterization and in vitro studies of doxorubicin-loaded magnetic nanoparticles grafted to smart copolymers on A549 lung cancer cell line. *Journal of Nanobiotechnology* **2012**, *10*, 46, doi:10.1186/1477-3155-10-46.
6. Dutta, S.; Parida, S.; Maiti, C.; Banerjee, R.; Mandal, M.; Dhara, D. Polymer grafted magnetic nanoparticles for delivery of anticancer drug at lower pH and elevated temperature. *Journal of Colloid and Interface Science* **2016**, *467*, 70-80, doi:https://doi.org/10.1016/j.jcis.2016.01.008.
7. Omidirad, R.; Rajabi Hosseinpour, F.; Farahani, B. Preparation and in vitro drug delivery response of doxorubicin loaded PAA coated magnetite nanoparticles. *Journal of the Serbian Chemical Society* **2013**, *78*, 1609-1616, doi:10.2298/jsc121225041o.
8. Le, T.T.H.; Bui, T.Q.; Ha, T.M.T.; Le, M.H.; Pham, H.N.; Ha, P.T. Optimizing the alginate coating layer of doxorubicin-loaded iron oxide nanoparticles for cancer hyperthermia and chemotherapy. *Journal of Materials Science* **2018**, *53*, 13826-13842, doi:10.1007/s10853-018-2574-z.
9. Yang, Y.; Guo, Q.; Peng, J.; Su, J.; Lu, X.; Zhao, Y.; Qian, Z. Doxorubicin-Conjugated Heparin-Coated Superparamagnetic Iron Oxide Nanoparticles for Combined Anticancer Drug Delivery and Magnetic Resonance Imaging. *Journal of Biomedical Nanotechnology* **2016**, *12*, 1963-1974, doi:10.1166/jbn.2016.2298.
10. Javid, A.; Ahmadian, S.; Saboury, A.A.; Kalantar, S.M.; Rezaei-Zarchi, S. Novel biodegradable heparin-coated nanocomposite system for targeted drug delivery. *RSC Advances* **2014**, *4*, 13719-13728, doi:10.1039/C3RA43967D.
11. Rouhollah, K.; Pelin, M.; Serap, Y.; Gozde, U.; Ufuk, G. Doxorubicin Loading, Release, and Stability of Polyamidoamine Dendrimer-Coated Magnetic Nanoparticles. *Journal of Pharmaceutical Sciences* **2013**, *102*, 1825-1835, doi:10.1002/jps.23524.
12. Chandra, S.; Mehta, S.; Nigam, S.; Bahadur, D. Dendritic magnetite nanocarriers for drug delivery applications. *New Journal of Chemistry* **2010**, *34*, 648-655, doi:10.1039/B9NJ00609E.
13. Javid, A.; Ahmadian, S.; Saboury, A.A.; Kalantar, S.M.; Rezaei-Zarchi, S. Chitosan-Coated Superparamagnetic Iron Oxide Nanoparticles for Doxorubicin Delivery: Synthesis and Anticancer Effect Against Human Ovarian Cancer Cells. *Chemical Biology & Drug Design* **2013**, *82*, 296-306, doi:https://doi.org/10.1111/cbdd.12145.

14. Altalhi, T.A.; Ibrahim, M.M.; Mersal, G.A.M.; Mahmoud, M.H.H.; Kumeria, T.; El-Desouky, M.G.; El-Bindary, A.A.; El-Bindary, M.A. Adsorption of doxorubicin hydrochloride onto thermally treated green adsorbent: Equilibrium, kinetic and thermodynamic studies. *Journal of Molecular Structure* **2022**, *1263*, 133160, doi:https://doi.org/10.1016/j.molstruc.2022.133160.
15. AlHazmi, G.A.A.; AbouMelha, K.S.; El-Desouky, M.G.; El-Bindary, A.A. Effective adsorption of doxorubicin hydrochloride on zirconium metal-organic framework: Equilibrium, kinetic and thermodynamic studies. *Journal of Molecular Structure* **2022**, *1258*, 132679, doi:https://doi.org/10.1016/j.molstruc.2022.132679.
16. Chakraborty, D.; Chauhan, P.; Kumar, S.; Chaudhary, S.; Chandrasekaran, N.; Mukherjee, A.; Ethiraj, K.R. Utilizing corona on functionalized selenium nanoparticles for loading and release of doxorubicin payload. *Journal of Molecular Liquids* **2019**, *296*, doi:10.1016/j.molliq.2019.111864.
17. Akturk, O. The anticancer activity of doxorubicin-loaded levan-functionalized gold nanoparticles synthesized by laser ablation. *Int J Biol Macromol* **2022**, *196*, 72-85, doi:10.1016/j.ijbiomac.2021.12.030.
18. Kurdyukov, D.A.; Eurov, D.A.; Shmakov, S.V.; Kirilenko, D.A.; Kukushkina, J.A.; Smirnov, A.N.; Yagovkina, M.A.; Klimenko, V.V.; Koniakhin, S.V.; Golubev, V.G. Fabrication of doxorubicin-loaded monodisperse spherical micro-mesoporous silicon particles for enhanced inhibition of cancer cell proliferation. *Microporous and Mesoporous Materials* **2019**, *281*, 1-8, doi:https://doi.org/10.1016/j.micromeso.2019.02.029.
19. Xu, G.; Zhang, W.; Du, J.; Yuan, X.; Zhang, W.; Yan, W.; Liu, G. Biomass-derived porous carbon with high drug adsorption capacity undergoes enzymatic and chemical degradation. *J Colloid Interface Sci* **2022**, *622*, 87-96, doi:10.1016/j.jcis.2022.04.064.
20. Demin, A.M.; Vakhrushev, A.V.; Valova, M.S.; Korolyova, M.A.; Uimin, M.A.; Minin, A.S.; Pozdina, V.A.; Byzov, I.V.; Tumashov, A.A.; Chistyakov, K.A.; et al. Effect of the Silica–Magnetite Nanocomposite Coating Functionalization on the Doxorubicin Sorption/Desorption. *Pharmaceutics* **2022**, *14*, doi:10.3390/pharmaceutics14112271.
21. Jiang, X.; Zhang, D.; Sun, R.; Wang, H.; Yang, Y.; Guo, H.; Tang, Y. A combined experimental and molecular dynamics simulation study on doxorubicin adsorption on strontium-substituted hydroxyapatite hollow microspheres. *Applied Surface Science* **2021**, *542*, 148667, doi:https://doi.org/10.1016/j.apsusc.2020.148667.
22. Singh, N.; Nayak, J.; Sahoo, S.K.; Kumar, R. Glutathione conjugated superparamagnetic Fe3O4-Au core shell nanoparticles for pH controlled release of DOX. *Materials Science and Engineering: C* **2019**, *100*, 453-465, doi:https://doi.org/10.1016/j.msec.2019.03.031.
23. Azlegini, A.; Javadpor, S.; Bahrolom, M. Liposome-Fe3O4-Doxorubicin Mediated Treatment of Melanoma Tumors. *Advanced Pharmaceutical Bulletin* **2022**, doi:10.34172/apb.2023.034.
24. Kovrigina, E.; Chubarov, A.; Dmitrienko, E. High Drug Capacity Doxorubicin-Loaded Iron Oxide Nanocomposites for Cancer Therapy. *Magnetochemistry* **2022**, *8*, doi:10.3390/magnetochemistry8050054.
25. Qi, C.; Wang, W.; Wang, P.; Cheng, H.; Wang, X.; Gong, B.; Xie, A.; Shen, Y. Facile Synthesis of Fe(3)O(4)@Au/PPy-DOX Nanoplatform with Enhanced Glutathione Depletion and Controllable Drug Delivery for Enhanced Cancer Therapeutic Efficacy. *Molecules (Basel, Switzerland)* **2022**, *27*, doi:10.3390/molecules27134003.
26. Nogueira, J.; Soares, S.F.; Amorim, C.O.; Amaral, J.S.; Silva, C.; Martel, F.; Trindade, T.; Daniel-da-Silva, A.L. Magnetic Driven Nanocarriers for pH-Responsive Doxorubicin Release in Cancer Therapy. *Molecules (Basel, Switzerland)* **2020**, *25*, doi:10.3390/molecules25020333.
27. Wang, Y.; Yang, S.T.; Wang, Y.; Liu, Y.; Wang, H. Adsorption and desorption of doxorubicin on oxidized carbon nanotubes. *Colloids Surf B Biointerfaces* **2012**, *97*, 62-69, doi:10.1016/j.colsurfb.2012.04.013.
28. Shafiei-Irannejad, V.; Rahimkhoei, V.; Molaparast, M.; Akbari, A. Synthesis and characterization of novel hybrid nanomaterials based on β-cyclodextrine grafted halloysite nanotubes for delivery of doxorubicin to MCF-7 cell line. *Journal of Molecular Structure* **2022**, *1262*, 133004, doi:https://doi.org/10.1016/j.molstruc.2022.133004.

29. Abdelfattah, A.; Aboutaleb, A.E.; Abdel‐Aal, A.B.M.; Abdellatif, A.A.H.; Tawfeek, H.M.; Abdel-Rahman, S.I. Design and optimization of PEGylated silver nanoparticles for efficient delivery of doxorubicin to cancer cells. *Journal of Drug Delivery Science and Technology* **2022**, *71*, 103347, doi:https://doi.org/10.1016/j.jddst.2022.103347.
30. Hernandes, E.P.; Bini, R.D.; Endo, K.M.; de Oliveira Junior, V.A.; de Almeida, I.V.; Dias, G.S.; dos Santos, I.A.; de Oliveira, P.N.; Vicentini, V.E.; Cotica, L.F. Doxorubicin-Loaded Magnetic Nanoparticles: Enhancement of Doxorubicin’s Effect on Breast Cancer Cells (MCF-7). *Magnetochemistry* **2022**, *8*, doi:10.3390/magnetochemistry8100114.
31. Abdelhalim, A.O.E.; Ageev, S.V.; Petrov, A.V.; Meshcheriakov, A.A.; Luttsev, M.D.; Vasina, L.V.; Nashchekina, I.A.; Murin, I.V.; Molchanov, O.E.; Maistrenko, D.N.; et al. Graphene oxide conjugated with doxorubicin: Synthesis, bioactivity, and biosafety. *Journal of Molecular Liquids* **2022**, *359*, 119156, doi:https://doi.org/10.1016/j.molliq.2022.119156.
32. Liu, X.; Wang, C.; Wang, X.; Tian, C.; Shen, Y.; Zhu, M. A dual-targeting Fe3O4@C/ZnO-DOX-FA nanoplatform with pH-responsive drug release and synergetic chemo-photothermal antitumor in vitro and in vivo. *Materials Science and Engineering: C* **2021**, *118*, 111455, doi:https://doi.org/10.1016/j.msec.2020.111455.
33. Eslami, P.; Albino, M.; Scavone, F.; Chiellini, F.; Morelli, A.; Baldi, G.; Cappiello, L.; Doumett, S.; Lorenzi, G.; Ravagli, C.; et al. Smart Magnetic Nanocarriers for Multi-Stimuli On-Demand Drug Delivery. *Nanomaterials* **2022**, *12*, doi:10.3390/nano12030303.
34. Cai, W.; Guo, M.; Weng, X.; Zhang, W.; Chen, Z. Adsorption of doxorubicin hydrochloride on glutaric anhydride functionalized Fe3O4@SiO2 magnetic nanoparticles. *Materials Science and Engineering: C* **2019**, *98*, 65-73, doi:https://doi.org/10.1016/j.msec.2018.12.145.
35. Abasalta, M.; Asefnejad, A.; Khorasani, M.T.; Saadatabadi, A.R.; Irani, M. Adsorption and sustained release of doxorubicin from N-carboxymethyl chitosan/polyvinyl alcohol/poly(ε-caprolactone) composite and core-shell nanofibers. *Journal of Drug Delivery Science and Technology* **2022**, *67*, 102937, doi:https://doi.org/10.1016/j.jddst.2021.102937.
36. Chapter 16 - Gravity Separation. In *Mineral Processing Design and Operations (Second Edition)*, Gupta, A., Yan, D., Eds.; Elsevier: Amsterdam, 2016; pp. 563-628.
